# Detrimental Effect of Fungal 60-kDa Heat Shock Protein on Experimental *Paracoccidioides brasiliensis* Infection

**DOI:** 10.1371/journal.pone.0162486

**Published:** 2016-09-06

**Authors:** Fabrício Freitas Fernandes, Leandro Licursi de Oliveira, Taise Natali Landgraf, Gabriela Peron, Marcelo Vieira Costa, Arlete A. M. Coelho-Castelo, Vânia L. D. Bonato, Maria-Cristina Roque-Barreira, Ademilson Panunto-Castelo

**Affiliations:** 1 Department of Cellular and Molecular Biology, Ribeirão Preto School of Medicine, University of São Paulo, Ribeirão Preto, SP, Brazil; 2 Department of General Biology, Federal University of Viçosa, Viçosa, MG, Brazil; 3 Department of Biochemistry and Immunology, School of Medicine of Ribeirão Preto, University of São Paulo, Ribeirão Preto, SP, Brazil; 4 Department of Structural and Functional Biology, Institute of Biology, State University of Campinas, Campinas, SP, Brazil; 5 Department of Biology, Ribeirão Preto Faculty of Philosophy, Sciences and Letters, University of São Paulo, Ribeirão Preto, SP, Brazil; University of Texas at San Antonio, UNITED STATES

## Abstract

The genus *Paracoccidioides* comprises species of dimorphic fungi that cause paracoccidioidomycosis (PCM), a systemic disease prevalent in Latin America. Here, we investigated whether administration of native 60-kDa heat shock protein of *P*. *brasiliensis* (nPbHsp60) or its recombinant counterpart (rPbHsp60) affected the course of experimental PCM. Mice were subcutaneously injected with nPbHsp60 or rPbHsp60 emulsified in complete’s Freund Adjuvant (CFA) at three weeks after intravenous injection of *P*. *brasiliensis* yeasts. Infected control mice were injected with CFA or isotonic saline solution alone. Thirty days after the nPbHsp60 or rPbHsp60 administration, mice showed remarkably increased fungal load, tissue inflammation, and granulomas in the lungs, liver, and spleen compared with control mice. Further, rPbHsp60 treatment *(i)* decreased the known protective effect of CFA against PCM and *(ii)* increased the concentrations of IL-17, TNF-α, IL-12, IFN-γ, IL-4, IL-10, and TGF-β in the lungs. Together, our results indicated that PbHsp60 induced a harmful immune response, exacerbated inflammation, and promoted fungal dissemination. Therefore, we propose that PbHsp60 contributes to the fungal pathogenesis.

## Introduction

The genus *Paracoccidioides* includes species of dimorphic fungi that cause paracoccidioidomycosis (PCM), a granulomatous systemic mycosis prevalent in Latin America [1–3]. *Paracoccidioides* fungi are thermally dimorphic and grow as hypha at the environmental temperature and as yeast at 35°C-37°C. Fungal morphologic transition is essential for establishing an infection because infective conidia or mycelial fragments are inhaled and converted to pathogenic yeast forms in the host lungs [4, 5]. Prevalence of PCM is higher in men in rural areas who are involved in activities related to the management of contaminated soil and plants [6]. *P*. *brasiliensis* may also affect immunocompromised individuals as an occasional opportunistic infection that results in severe clinical manifestations and a high mortality rate [7]. Fungal invasion of host tissues induces inflammation characterized by macrophage activation and granuloma formation, which controls the dissemination of *Paracoccidioides* yeasts to other organs [8].

Clinical manifestations of *Paracoccidioides* infection may be directly associated with fungal factors, such as virulence and pathogenicity [9], or are related to host factors, such as genetic susceptibility and immune competence [10, 11]. However, fungal and host factors are intricately connected to each other because disease outcome depends on the interaction of fungal components with receptors on host phagocytic cells [12]. For this reason, strains of *P*. *brasiliensis*, which is the most studied species of the genus *Paracoccidioides*, have variable degrees of virulence and, consequently, can induce different host responses [13]. Interestingly, Bonfim et al. [13] described that less virulent *P*. *brasiliensis* strain is preferably recognized by receptors dectin-1 and Toll-like receptor (TLR) 2 present on innate immune cells and induce the production of balanced amounts of TNF-α and IL-10. On the other hand, most virulent *P*. *brasiliensis* strain promotes the production of TNF-α but not IL-10. These observations suggest that less virulent *P*. *brasiliensis* strains induce a more controlled response because IL-10, an anti-inflammatory cytokine, prevents host tissue injury that may result from TNF-α activity [13].

Several studies have identified and characterized components of *Paracoccidioides* fungi involved in their infection and pathogenicity to better understand their biology and interactions with host cells and to identify potential vaccine targets. Lipids, polysaccharides, and proteins were already mentioned as able to increase fungal pathogenicity [14, 15]. Studies with plasmid DNA cloned with the gene of heat shock protein (Hsp) of 65-kDa from *Mycobacterium leprae* [16] and with the gene encoding the P10 peptide from gp43 [17] have shown therapeutic effects in experimental PCM. *P*. *brasiliensis* proteins are the most studied because of their high immunogenicity. For example, gp43, which is the most studied component of *P*. *brasiliensis* yeasts, has significant potential for application in vaccine development or immunotherapy against PCM [15]. Also, a 27-kDa component of *P*. *brasiliensis* is under investigation for its use in the prophylaxis and treatment of PCM [18]. The rPb27 and rPb40, in addition to fluconazole chemotherapy, showed an additive protective effect [19, 20]. Moreover, the *P*. *brasiliensis* lectin, paracoccin, by interacting with TLR2 N-glycans on host cells, establishes protective responses against PCM [21].

To identify *P*. *brasiliensis* antigens that contributed to its pathogenicity and that could serve as potential vaccines or therapeutic targets, we examined *P*. *brasiliensis* components that bind to immobilized fetuin. Interestingly, the major component of the fetuin-bound fraction was identified as the heat shock protein of *P*. *brasiliensis* and the preparation was designed as nPbHsp60. Its administration or the administration of its recombinant counterpart (rPbHsp60) induced detrimental effects in *P*. *brasiliensis*-infected mice. Therefore, we propose that PbHsp60 contributes to the fungal pathogenesis.

## Materials and Methods

### Mice and Ethics Statement

This study was conducted in accordance with the ethical principles of animal research adopted by the Brazilian Society of Laboratory Animal Science and was approved by the Ethics Committee on Animal Use of the Ribeirão Preto Medical School, USP (protocol: 146/2007). Male BALB/c mice between 6–8 weeks of age and weighing 20–25 g (n = 5/group) were obtained from the Animal Facility of Ribeirão Preto Campus and were maintained at the Animal Facility of Ribeirão Preto School of Medicine, University of São Paulo (USP). They were acclimated to the facility for one week prior to initiating the experiment, housed in individually ventilated cages, light-tight cabinets (Alesco, Capivari, Brazil), maintained at 20–22°C, a 12 h light-dark cycle, and with access to chow and water ad libitum. All cages were cleaned twice a week and bedded with autoclaved soft wood shavings.

### Fungal Isolate

Yeast cells of a highly virulent *P*. *brasiliensis* strain (Pb18) were cultured on YPD broth (1% yeast extract, 2% peptone, and 2% dextrose) and were incubated at 36°C for 7 days. Virulence and viability of the yeast cells were maintained as described previously [22, 23]. Briefly, the virulence was maintained by constant mice infections and recovery of Pb18 strain. The viability was performed by the fluorescein diacetate-ethidium bromide treatment Only suspensions containing >90% viable cells were used for infecting mice.

### Antigen Preparation

Cultured yeast cells were harvested by centrifugation at 7,000 × *g* at 4°C for 10 minutes, washed with 10 mM phosphate-buffered saline (PBS), pH 7.2, and disrupted by sonication on ice (five cycles of 1 minute each) at 200 W (Unique UltraSonic Mixing, mod. DES 500, 4 mm probe; Unique Group, Indaiatuba, Brazil), followed by centrifugation at 7,000 × *g* at 4°C for 10 minutes. Supernatant containing *P*. *brasiliensis* soluble antigens (PbAgs) was filtered through a 0.22-μm filter (Millipore, Billerica, USA). Approximately 5 mg PbAgs were resolved by performing chromatography with a 5-mL fetuin—agarose column (Sigma Chemical Co., St. Louis, USA) that was previously equilibrated with 20 mM sodium phosphate buffer (pH 7.4). After washing with 10 column volumes (cv) of the equilibrating buffer, the column was sequentially eluted using 5 cv of 0.4 M d-glucose, d-mannose, d-galactose, or α-lactose in PBS or with 1 M NaCl in PBS. The eluted fractions were dialyzed against water by using centrifugal filtration devices with a molecular weight cut-off of 10,000 kDa (Millipore). Concentration of proteins eluted with 1 M NaCl was determined using a BCA kit (Pierce Chemical Co., Rockford, USA).

### Electrophoresis and Protein Identification

Eluted protein and rPbHsp60 (~5 μg) were resuspended in 5× loading buffer (500 mM Tris-HCl [pH 6.5], 2.5% SDS, 10% glycerol, 2.5% β-mercaptoethanol, and 0.1% bromophenol blue) and were heated at 100°C for 3 minutes. Next, the preparations were resolved by performing sodium dodecyl sulfate-polyacrylamide gel electrophoresis (SDS-PAGE) on a 12.5% by using Mini-Protean Tetra System (Bio-Rad Laboratories, Richmond, USA). The gels were stained with Coomassie brilliant blue G250 (USB Corporation, Cleveland, USA). Proteins with known molecular masses were used as standards (LMW-SDS Marker Kit; GE Healthcare UK Ltd, Buckinghamshire, England). A semi-quantitative analysis of the relative amount of 68-kDa protein band was carried out by converting the density of protein bands in the gel picture using the ImageJ 1.37v software (National Institutes of Health, Bethesda, USA) as the percent of the total gel density. Next, 60-kDa bands from column-eluted fraction and rPbHsp60 were excised from the gel, were digested *in situ* with trypsin. Peptides were extracted from gel and dried in SpeedVac, resuspended in 50 μl 1% formic acid, centrifuged and transferred to HPLC vial. Ten μl sample was typically analyzed on the system. All mass spectrometry analyses were performed on an Agilent 6520 Q-TOF mass spectrometer equipped with an Agilent 1200 series liquid chromatograph and an Agilent Chip Cube LC-MS interface (1D nLC-MS-MS) at the FingerPrint Proteomics and Mass Spectrometry Facility, College of Life Sciences, University of Dundee. Mascot (version 2.3; Matrix, United Kingdom) analysis was performed to identify peptides and to search for proteins in the NCBI nonredundant (nr) database. The identified peptides were searched against NCBInr *E*. *coli* database to find possible bacterial protein contaminants in the sample.

### Cloning of *P*. *brasiliensis* cDNA Encoding PbHsp60

Total RNA was extracted from *P*. *brasiliensis* yeast culture by using Trizol (Life Technologies, Carslbad, USA), according to the manufacturer's protocol. Reverse transcription-PCR (RT-PCR) was performed using oligo-dT_12-18_ primer (Life Technology) and SuperScript II Reverse Transcriptase (Life Technologies) for synthesizing cDNA. To amplify the cDNA region encoding PbHsp60 were used the oligonucleotide primers 5′-CGAATTCATGATGCAGCGAGCTTTTACTTCCT-3′ (sense) and 5′-CTCGAGGAACATACCCCCGCCCATAC-3′ (antisense) and high-fidelity *Taq* polymerase (Life Technologies). The amplified fragment was cloned into pGEM-T vector (Promega, Madison, USA) and was sequenced at the Center for Human Genome Studies, Institute of Biosciences, USP. Next, the fragment was removed from the pGEM-T vector by using EcoRI and XhoI and was subcloned into pET28a vector (Novagen, San Diego, USA).

### Expression and Purification of rPbHsp60 in *Escherichia coli*

*E*. *coli* transformed with pET28a–*HSP60* vector were grown in LB medium (3 L) supplemented with kanamycin sulfate (50 μg/mL) in a shaking incubator at 180 rpm and 37°C until optical density at 600 nm reached 0.5. Next, 0.4 mM isopropyl-β-d-thiogalactopyranoside was added to the culture medium to induce the expression of the recombinant protein. After 6 hours, bacterial cells were harvested by centrifugation at 3,000 × g, were resuspended in a buffer (50 mM NaH_2_PO_4_, 300 mM NaCl, and 30 mM imidazole [pH 8.0]), and were lysed by sonication. Lipopolysaccharide and other bacterial contaminants were removed from the sample by washing it at least five times with a buffer containing 50 mM NaH_2_PO_4_, 300 mM NaCl, 2 M urea, 5 mM 2-mercaptoethanol, and 0.5% Triton X-100 (pH 8.0) through centrifugation at 10,000 × *g* [24]. Pellet containing the insoluble fraction (inclusions bodies) was resuspended in a denaturing solubilization buffer (50 mM NaH_2_PO_4_, 300 mM NaCl, 30 mM imidazole, 7 M urea, 5 mM 2-mercaptoethanol, and 0.5% Tween 20 [pH 8.0]) and was incubated for 1 hour at room temperature. The denatured material was recovered by centrifugation at 10,000 × *g* and was filtered through Millex-GV PVDF (pore size, 0.22 μm; Millipore). The recombinant protein was purified by performing metal chelate affinity chromatography with a Ni^2+^–Sepharose affinity column (His-Trap; GE Healthcare). Next, the recombinant protein was eluted with elution buffer (50 mM NaH_2_PO_4_, 300 mM NaCl, 250 mM imidazole, 7 M urea, and 5 mM 2-mercaptoethanol), refolded by dialysis against PBS, and concentrated by ultrafiltration. Protein concentration was determined using Coomassie Plus (Bradford) Assay Kit (Pierce Chemical Co.). Purity, size, and identity of the recombinant protein were evaluated using SDS-PAGE and 1D nLC-MS-MS, similar to those described above for column-eluted fraction. The rPbHsp60 preparation contained less than 0.05 ng/mL of bacterial endotoxin, as determined by the *Limulus amoebocyte* lysate assay (Sigma Chemical Co.).

### Experimental Treatment Protocol

Mice were intravenously inoculated by retro-orbital plexus with 1 × 10^6^ viable yeast cells and daily monitored for signs of distress or illness, and mortality. None of them became seriously ill or died prior to the experimental endpoint. On day 21 postinfection, the mice were divided into groups containing five animals each. In experiments involving nPbHsp60, the mice were subcutaneously injected with a single 100 μL dose of one of the following preparations: (1) 25 μg nPbHsp60 in PBS, (2) 25 μg nPbHsp60 in PBS and emulsified in CFA (Sigma Chemical Co.) (nPbHsp60 + CFA, 1:1), (3) PBS emulsified in CFA, and (4) only PBS. In the experiments with recombinant protein, 50 μg of rPbHsp60 were used. On day 30 after the treatment, mice were anesthetized by intraperitoneal injection of ketamine (100 mg/kg) and xylazine (8 mg/kg) mixture and submitted to euthanasia by cervical dislocation. Their lungs, livers, and spleens were removed aseptically for performing histopathological analyses and for quantifying fungal load and cytokine levels.

### CFU, Tissue Injury and Cytokine Profile Evaluation

The right lungs and half portions of the livers and spleens were weighed and homogenized in 1 mL sterile PBS by using a tissue homogenizer (Ultra-Turrax T25 Basic; IKA Works, Inc., Wilmington, USA). Colony-forming units (CFU) of *P*. *brasiliensis* yeast cells were determined as described previously [23, 25]. For determining cytokine levels, the organ homogenates were centrifuged at 5,000 × *g* for 10 minutes and their supernatants were stored at -20°C.

The left lungs and approximately half portions of the livers and spleens were fixed in 10% neutral buffered formalin for 24 hours and were embedded in paraffin. Next, the tissue sections (thickness, 5 μm) were stained with hematoxylin and eosin (H&E) by using standard protocols and were analyzed by performing light microscopy with Axiophot photomicroscope (Carl Zeiss, Jena, Germany) coupled with JVC TK-1270 camera (Victor Company of Japan Ltd, Tokyo, Japan). Total area of the lung sections and inflammatory infiltrates of *P*. *brasiliensis* yeasts per lung section were measured using a computer-aided image analysis software (ImageJ 1.37v; National Institutes of Health, Bethesda, USA).

Concentrations of IL-17, IFN-γ, TNF-α, IL-4, IL-10, IL-12, and TGF-β in the lung homogenates were measured by performing capture ELISA with OptEIA ELISA sets (BD PharMingen, San Diego, USA), according to the manufacturer's protocol. Cytokine concentrations were determined by referring to a standard curve for serial two-fold dilutions of recombinant murine cytokines.

### Statistical Analysis

Statistical differences among means of different experimental groups were determined using one-way analysis of variance followed by Bonferroni's post-test. Differences were considered statistically significant at *P* < 0.05. All the experiments were performed at least three times.

## Results

### Isolation and Identification of 60 kDa Protein Isolated from PbAgs

**Fig 1:**
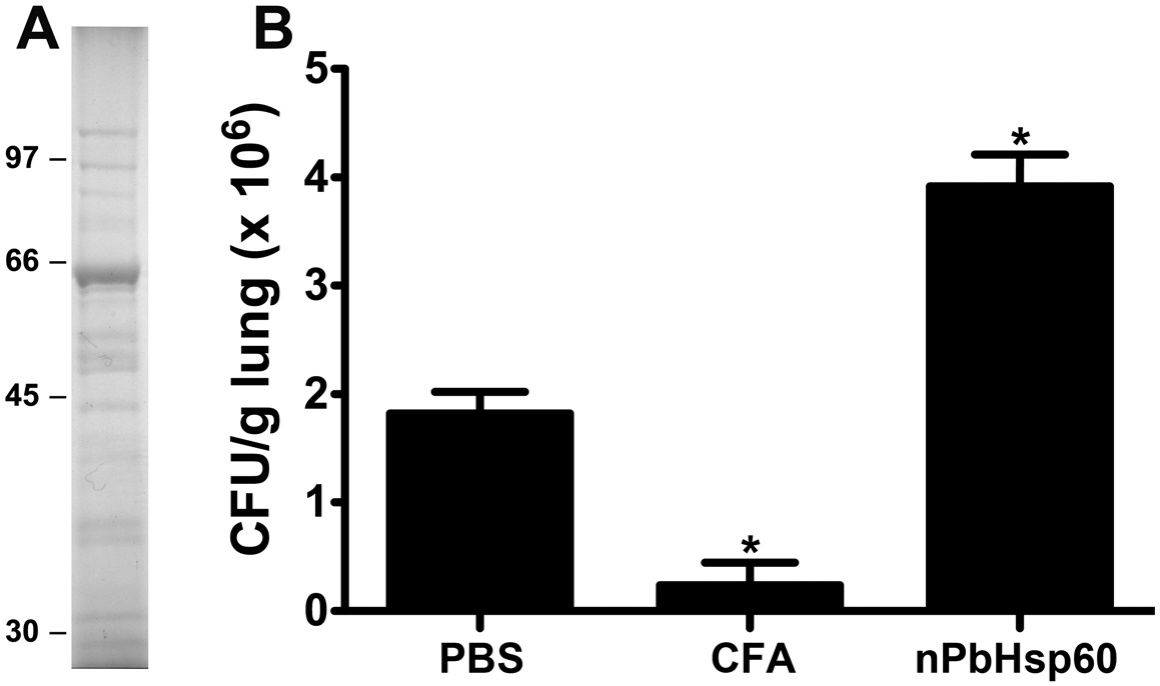
Native PbHsp60 increase fungal load in *P*. *brasiliensis*-infected mice. (A) Isolation of nPbHsp60 from *P*. *brasiliensis* by using a fetuin—agarose column equilibrated with 20 mM sodium phosphate buffer (pH 7.4). Chromatography was monitored spectrophotometrically at 280 nm. The fraction eluted with 1 M NaCl was concentrated, dialyzed against 20 mM sodium phosphate buffer (pH 7.4), and analyzed by performing SDS-PAGE with a 12.5% gel. The gel was stained with Coomassie brilliant blue. Migration positions of molecular mass markers are shown on MW in kDa. (B) Mice injected with 1 × 10^6^ *P*. *brasiliensis* yeast cells were treated with or without CFA or nPbHsp60 on day 21 postinfection. Lung homogenates were obtained from these mice on day 30 after the treatment and were analyzed for the CFU of *P*. *brasiliensis* yeast. Data are expressed as the mean ± standard deviation of five mice per group; *P < 0.05 compared to the other groups.

A preparation of PbAgs was affinity chromatographed on an immobilized fetuin column. The bound proteins could not be eluted using carbohydrate solutions (0.4 M d-glucose, d-mannose, d-galactose, or α-lactose). Otherwise, protein elution was achieved by using 1 M NaCl solution in PBS, a fraction containing a 60-kDa major band on SDS-PAGE (Fig 1A) that comprised 68% of all proteins in the sample. The 60-kDa protein band was digested with trypsin and the one dimensional nano-LC-MS/MS analysis of tryptic fragments revealed that 141 peptides matched the sequence of PbHsp60 (GenBank accession number: XP_010763632.1), resulting in a sequence coverage of 43% (S1 Fig and S1 Table). Because Hsp are protein highly conserved across the species, when we blasted the MS peptide sequences against the *E*. *coli* database, no correlation with *E coli* GroEL or other proteins was found. Therefore, this enriched preparation of PbHsp60 was designed nPbHsp60, which was used, as indicated, in subsequent experiments.

### Detrimental Effects of nPbHsp60 Administration to Mice with Experimental PCM

In initial experiments, we evaluated the effect of nPbHsp60 treatment on the course of experimental PCM in mice. Mice infected with *P*. *brasiliensis* yeast cells for 3 weeks were injected with nPbHsp60 or control preparations. The nPbHsp60-treated mice showed increased fungal load compared with PBS- or CFA-treated mice (Fig 1B). Treatment with CFA, an efficient inducer of Th1 response in *P*. *brasiliensis* infection [23], remarkably decreased the CFUs of *P*. *brasiliensis* yeasts in the lungs compared with treatment with PBS (Fig 1B).

**Fig 2:**
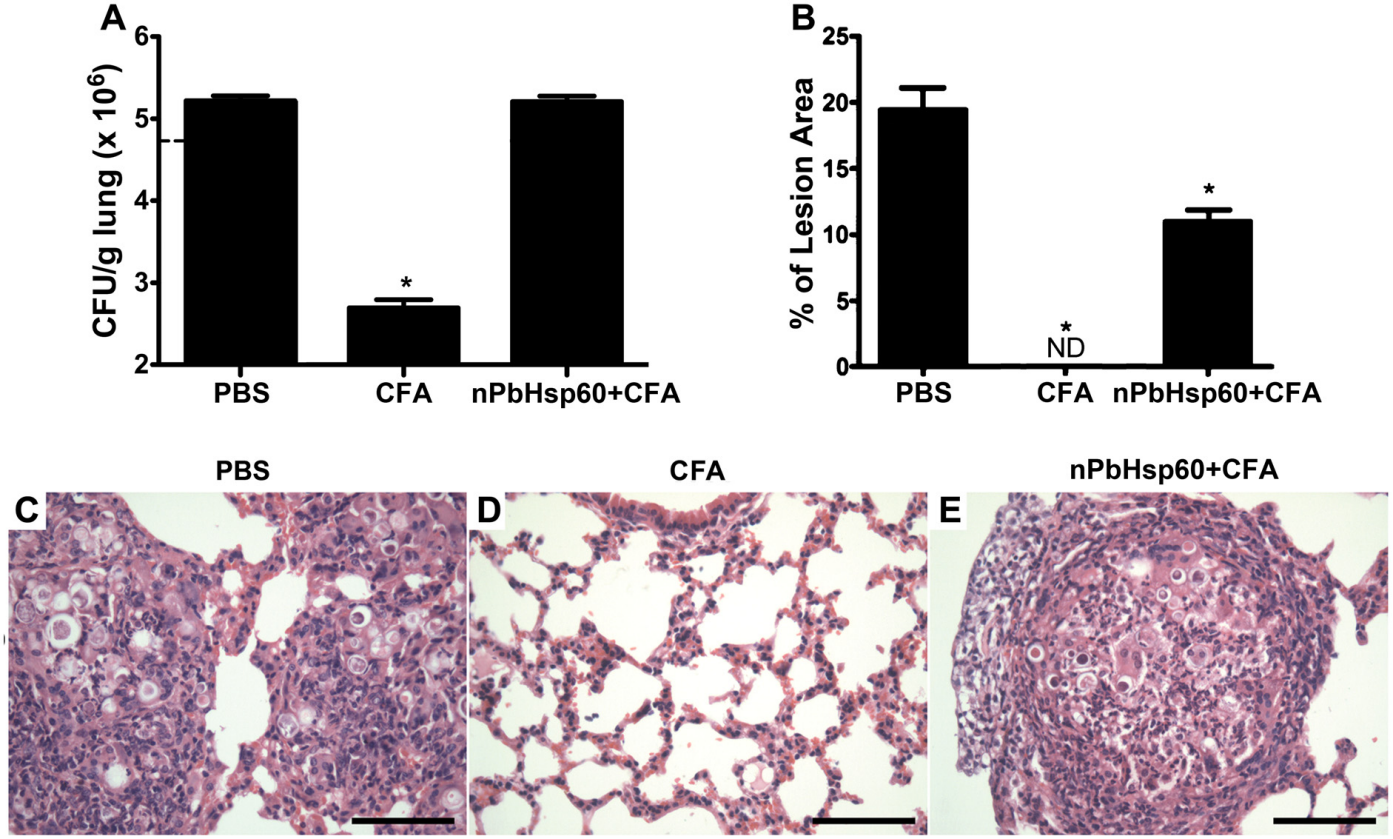
Native PbHsp60 reverse the beneficial effect of CFA on experimental PCM. Mice injected with 1 × 10^6^ *P*. *brasiliensis* yeast cells were treated with PBS, CFA, or CFA-emulsified nPbHsp60 (nPbHsp60 + CFA) on day 21 postinfection. (A) Lung homogenates were obtained from these mice on day 30 after the treatment and were analyzed for the CFU of *P*. *brasiliensis* yeast cells. (B–E) Lung tissues obtained on day 30 after the treatment were fixed in formalin, embedded in paraffin, cut into 5-μm sections, stained with H&E, and analyzed by light microscopy. Scale bars indicate 200 μm. (B) Morphometric analyses were performed using lung sections obtained from *P*. *brasiliensis*-infected mice treated with (C) PBS, (D) CFA, or (E) nPbHsp60 + CFA. Percentage lesion area was measured using a computer-aided image analysis software. Data are expressed as mean ± standard deviation; ND, not detected; *P < 0.05 compared with the other groups.

Next, we determined whether the administration of nPbHsp60 changed the beneficial effects of CFA-treatment. Intriguingly, when the *P*. *brasiliensis*-infected mice were treated with nPbHsp60 (25 μg) emulsified in CFA (nPbHsp60 + CFA), they had numbers of CFU on day 30 after treatment, at least, 2-fold higher than those detected in CFA-treated mice and quite similar to those in the lung from PBS-treated mice (infection control) (Fig 2A). These results indicate that the CFA effect in restraining fungal growth was decreased by nPbHsp60. Moreover, histological analysis showed an extensive lesion area (Fig 2B) with numerous granulomas containing high yeast load (Fig 2E) in the lungs of nPbHsp60 + CFA-treated and negative control mice (Fig 2C). In contrast, analysis of lung sections of *P*. *brasiliensis*-infected mice treated with CFA alone showed well-preserved bronchoalveolar architecture, with no detectable granulomas or yeast cells (Fig 2D). Moreover, no granulomas and yeast cells were detected in the livers and spleens of these mice. Together, these results supported the hypothesis that nPbHsp60 treatment impaired the beneficial effect of CFA against PCM.

### rPbHsp60 Reproduces the nPbHsp60 Effects on the Experimental PCM Course

**Fig 3:**
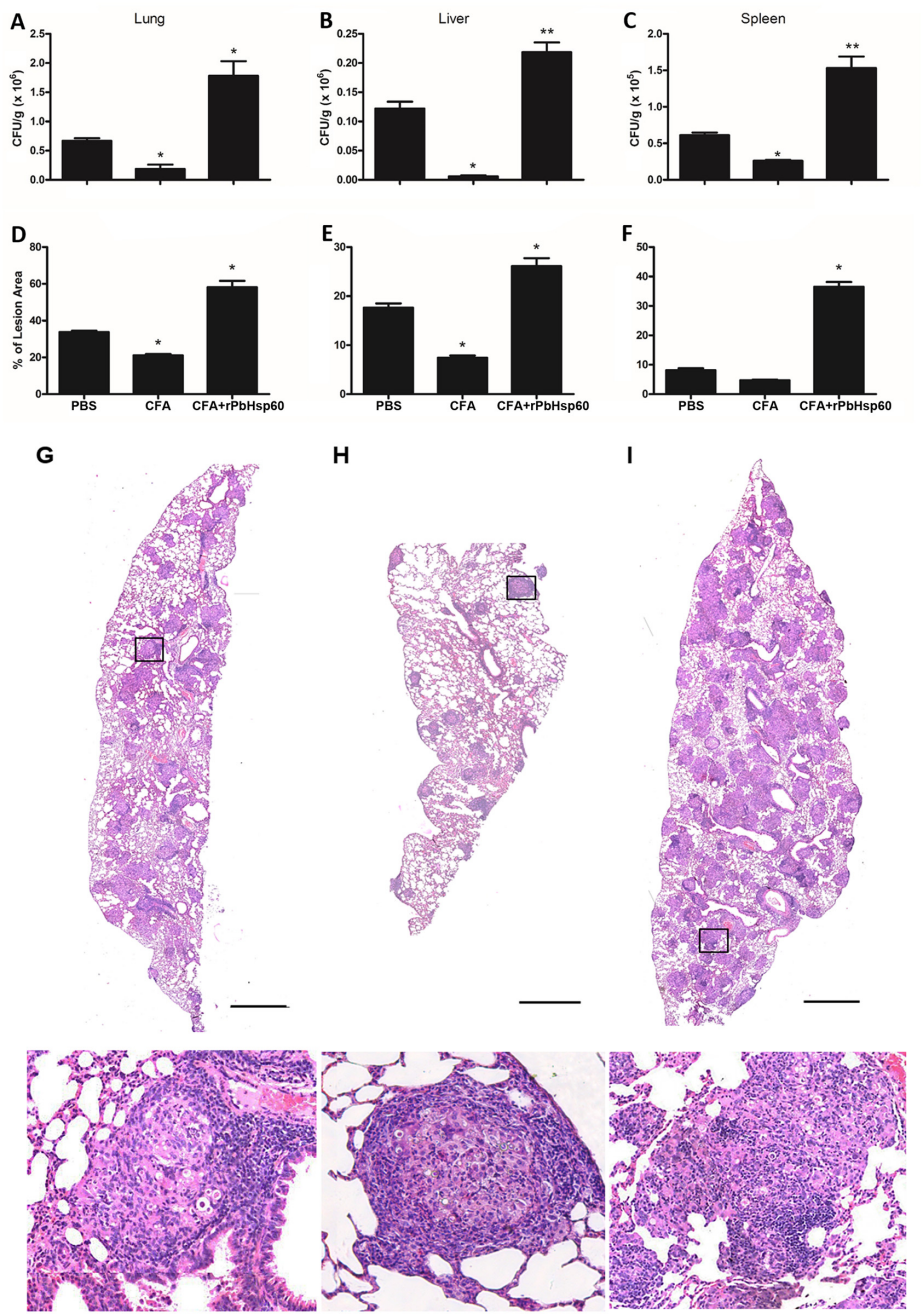
Recombinant PbHsp60 disrupts the beneficial effect of CFA on experimental PCM. Mice inoculated with 1 x 10^6^ *P*. *brasiliensis* yeast cells were treated, on day 21 postinfection, with PBS, CFA, or CFA emulsified rPbHsp60 (rPbHsp60 + CFA; 50 μg). Thirty days after the treatment animals were sacrificed and lung (A), liver (B), and spleen (C) homogenates were analyzed for the CFU of *P*. *brasiliensis* yeast cells. Data are expressed as the mean ± standard deviation of five mice per group obtained from three independent experiments. Sections (5-μm) of these organs were stained with H&E and microscopically analyzed for the extension of granulomatous lesions using Image J software (panels D-F). Bars represent the mean ± standard deviation of percentage lesion areas; *P < 0.05 compared with the other groups. Panels G to I show representative images captured from the pulmonary tissue of mice of each experimental group: PBS-treated (G), CFA (H) and rPbHsp60 + CFA (I). Scale bars of the lung sections indicate 1 mm. Images in the bottom panel correspond to black squares indicated on the upper panels.

To validate our presumption that PbHsp60 was responsible for the activities exerted by the fetuin-bound fraction, we expressed rPbHsp60 in pET-28a–*HSP60*-transformed *E*. *coli* cells and purified it by performing His-Trap chromatography. We analyzed the obtained protein by SDS-PAGE, which produced a single band with apparent molecular mass of 60-kDa. MS analysis confirmed that the recombinant protein was PbHsp60. No correlation with *E coli* proteins was found when we blasted the MS peptide sequences against the *E*. *coli* database. This rPbHsp60 was biologically assayed by using the protocols adopted for experiments performed using nPbHsp60. At 51 days post-infection, i.e., 30 days after treatment of the *P*. *brasiliensis*-infected mice, we compared the fungal load and inflammation in the lungs, liver, and spleen of mice that received rPbHsp60 + CFA as treatment with those treated with CFA or PBS (Fig 3A–3C). As expected, CFA treatment decreased the fungal load compared with PBS, whereas rPbHsp60 + CFA duplicated the fungal load the examined organs, indicating that infection dissemination was lower in animals treated with CFA alone and maximum in mice injected with rPbHsp60 + CFA. Consistently, histopathological analysis showed that rPbHsp60 + CFA-injected mice had tissue injury more pronounced than CFA- or PBS-treated mice, an observation that was certified by the morphometric analysis of granulomatous lesions in the lung, liver, and spleen sections of the infected animals: the lesions occupied areas at least 50% more extended of the organs from rPbHsp60 + CFA-treated mice (Fig 3D–3F). Details of the pulmonary histology of the three groups of infected mice can be seen in Fig 3, Panels G-I. The comparative analysis of these panels shows that treatment with rPbHsp60 + CFA resulted in the formation of numerous loose granulomas with inflammation foci and dissemination of yeast cells in the lungs, whereas the CFA-treated mice displayed the most compact and well-structured granulomas (Fig 3H).

### rPbHsp60 Administration Increases the Production of Cytokines in the Lungs of *P*. *brasiliensis* Infected Mice

**Fig 4:**
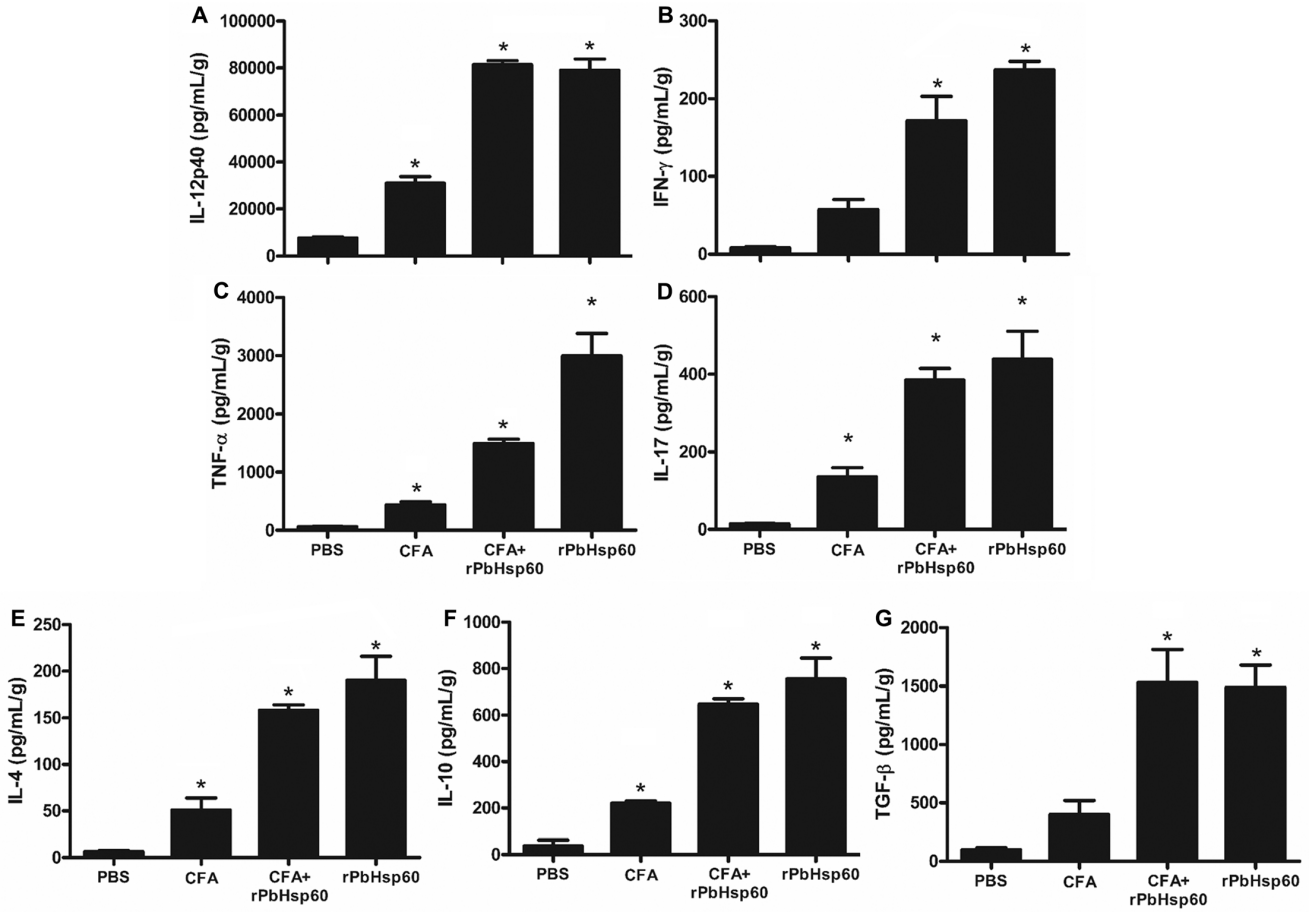
Treatment of *P*. *brasiliensis*-infected mice with rPbHsp60 emulsified or not in CFA increases the concentration of all the tested cytokines. Mice injected with 1 × 10^6^ *P*. *brasiliensis* yeast cells were treated with **PBS**, **CFA**, rPbHsp60 alone (**rPbHsp60**) or emulsified in CFA (**PbHsp60 + CFA**) on day 21 postinfection. Lung homogenates obtained from these mice on day 30 after the treatment were analyzed for the concentrations of (A) IL-12, (B) IFN-γ, (C) TNF-α, (D) IL-17, (E) IL-4, (F) IL-10, and (G) TGF-β. Data are expressed as the mean ± standard deviation of five mice per group obtained from three independent experiments; *P < 0.05 compared with the other groups.

We next investigated whether increased fungal load and pulmonary lesions in rPbHsp60 alone or rPbHsp60 + CFA-treated mice were associated with an unfavorable pattern of cytokine production in the lungs. Compared with CFA- or PBS-treated mice, the lungs of rPbHsp60 alone or rPbHsp60 + CFA-injected mice showed higher concentrations of different cytokines, including those of inflammatory (IL-17 and TNF-α), Th1 (IL-12 and IFN-γ), Th2 (IL-4), and regulatory (IL-10 and TGF-β) profiles (Fig 4). Notably, the most remarkable increase concerned the concentrations of proinflammatory cytokines IL-17, TNF-α, and IFN-γ, a condition that was consistent with severe inflammation and high fungal load observed in the lungs of rPbHsp60-treated mice (Fig 4).

## Discussion

To find components of *P*. *brasiliensis* yeasts that might affect the outcome of PCM, we isolated an enriched fraction of PbHsp60, which had detrimental effects when administrated to *P*. *brasiliensis*-infected mice. The recombinant counterpart of PbHsp60 was prepared and administered to infected mice and reproduced the detrimental effects of the native preparation. In both cases, the treatment resulted in increased fungal load and disseminated disease, as well as in severe pulmonary inflammatory lesions. Furthermore, nPbHsp60 and rPbHsp60 antagonized the beneficial effects of CFA, a known inducer of Th1 immunity that confers protection against PCM [23]. Detection of high pulmonary concentrations of several cytokines, particularly inflammatory cytokines, suggested that aggravation of PCM resulted from a dysregulated immune response induced by rPbHsp60 administration.

Hsps are evolutionarily well-conserved proteins that function as molecular chaperones. Hsps play key roles in cellular homeostasis; participate in protein folding, unfolding, and assembly; and prevent protein aggregation and denaturation [26]. Although existing in basal conditions with their housekeeping functions in the cells, Hsps have their expression upregulated and are also fundamental to cell survival under stress, such as high temperatures, toxins, and oxidative conditions [27]. In dimorphic fungi, including *P*. *brasiliensis*, Hsps expression is increased during the conidia to yeast transition [28, 29], an event that may be important in the fungal pathogenesis when conidia reach the lungs of hosts [30]. In the characterization studies of Hsp60 from *P*. *brasiliensis*, the authors showed that this protein was overexpressed during morphological transition [31] and was recognized by sera from humans with PCM [31, 32]. Besides heat shock, host systems exert additional stress by inducing immune response to prevent fungal colonization and tissue invasion, suggesting that Hsps are required for promoting fungal survival within hosts. This also suggests that Hsps are one of the main targets of immune response [33].

Because fungal Hsp60s are immunodominant antigens that trigger strong cellular and humoral immune responses [27, 34, 35], they are interesting therapeutic and vaccinal targets [36]. Soares et al. [37] reported that rPbHsp60 may serve as an efficient vaccine component for treating experimental PCM. The differences between our results and those of Soares et al. [37] may be due to the different infection routes and protocols for the administration of recombinant PbHsp60. While Soares et al. [37] used intranasal infection, we performed intravenous infection, which mimics a disseminated form of the disease and advantageously constitutes a model for preclinical therapeutic trials [38]. Furthermore, we administrated rPbHsp60 in a therapy regimen, whereas Soares et al. [37] adopted a prophylactic regimen. The protocol used in the present study was designed on the basis of a previous study where treatment of *P*. *brasiliensis*-infected mice with CFA decreased fungal load in up to 2-log and substantially increased the concentrations of protective cytokines [23]. In the present study, treatment of *P*. *brasiliensis*-infected mice with rPbHsp60 + CFA increased fungal load, number of diffuse granulomas with inflammation foci, and levels of all cytokines in lungs compared with that in *P*. *brasiliensis*-infected mice treated with CFA alone. Increased concentrations of proinflammatory cytokines (IL-17, TNF-α and IFN-γ) after rPbHsp60 treatment of *P*. *brasiliensis*-infected mice aggravated PCM, leading to severe inflammation, host tissue damage, impaired granuloma formation, and *P*. *brasiliensis* dissemination. Although anti-inflammatory cytokines IL-10 and TGF-β prevent inflammatory damage [39], lack of equilibrium in cytokine concentrations may favor tissue destruction. The risks of dysregulated cytokine production are clearly noted in other diseases, as septic shock syndrome [40, 41], which has a high mortality rate in humans [42]. Though anti-inflammatory cytokines are produced to compensate the high levels inflammatory response in patients with septic shock syndrome, they may be harmful, leading to cutaneous anergy, reduction of lymphocytes, decreased of monocytes response to cytokine stimulation, as well as decrease of human leukocytes antigens on monocytes [43]. Like in sepsis, an excessive cytokine production was harmful in *P*. *brasiliensis*-infected mice treated with PbHsp60, since that dysregulation may have been responsible, at the same time, for increased inflammation and tissue damage and decreased protective immune response against to *P*. *brasiliensis*, increasing the number of yeast in the tissue. Indeed, a fine-tuning between resistance and tolerance may explain the aspects of fungal infection, such as immunopathology and persistence [44].

Our study provides evidences that administered rPbHsp60 accounts for severe lesions in PCM and allows us to propose that PbHsp60 contributes to the fungal pathogenesis. Moreover, results of this study provide a perspective for controlling exacerbated immune responses and for designing new treatment approaches to induce appropriate immune response against deep mycosis.

## Supporting Information

S1 Fig MS/MS Mascot search data of the 60-kDa protein from *P*. *brasiliensis*. The 60-kDa band from *P*. *brasiliensis* was excised from the electrophoresis gel and digested *in situ* with trypsin. Peptides were extracted from gel and dried, resuspended in 50 μl 1% formic acid, centrifuged and transferred to HPLC vial. Spectrometry analyses were performed on an Agilent 6520 Q-TOF mass spectrometer equipped with an Agilent 1200 series liquid chromatograph and an Agilent Chip Cube LC-MS interface (1D nLC-MS-MS). Mascot (version 2.3; Matrix, United Kingdom) analysis was performed to identify peptides and to search for proteins in the NCBI nonredundant (nr) database.
(TIF)

S1 Table List of all proteins matched with at least one peptide with a significant score. (DOCX)
