# Supplementary figures and images for: Detrimental Effect of Fungal 60-kDa Heat Shock Protein on Experimental Paracoccidioides brasiliensis Infection
Source: PLoS One. 2016 Sep 6;11(9):e0162486. doi: 10.1371/journal.pone.0162486 (PMC5012565; doi:10.1371/journal.pone.0162486)

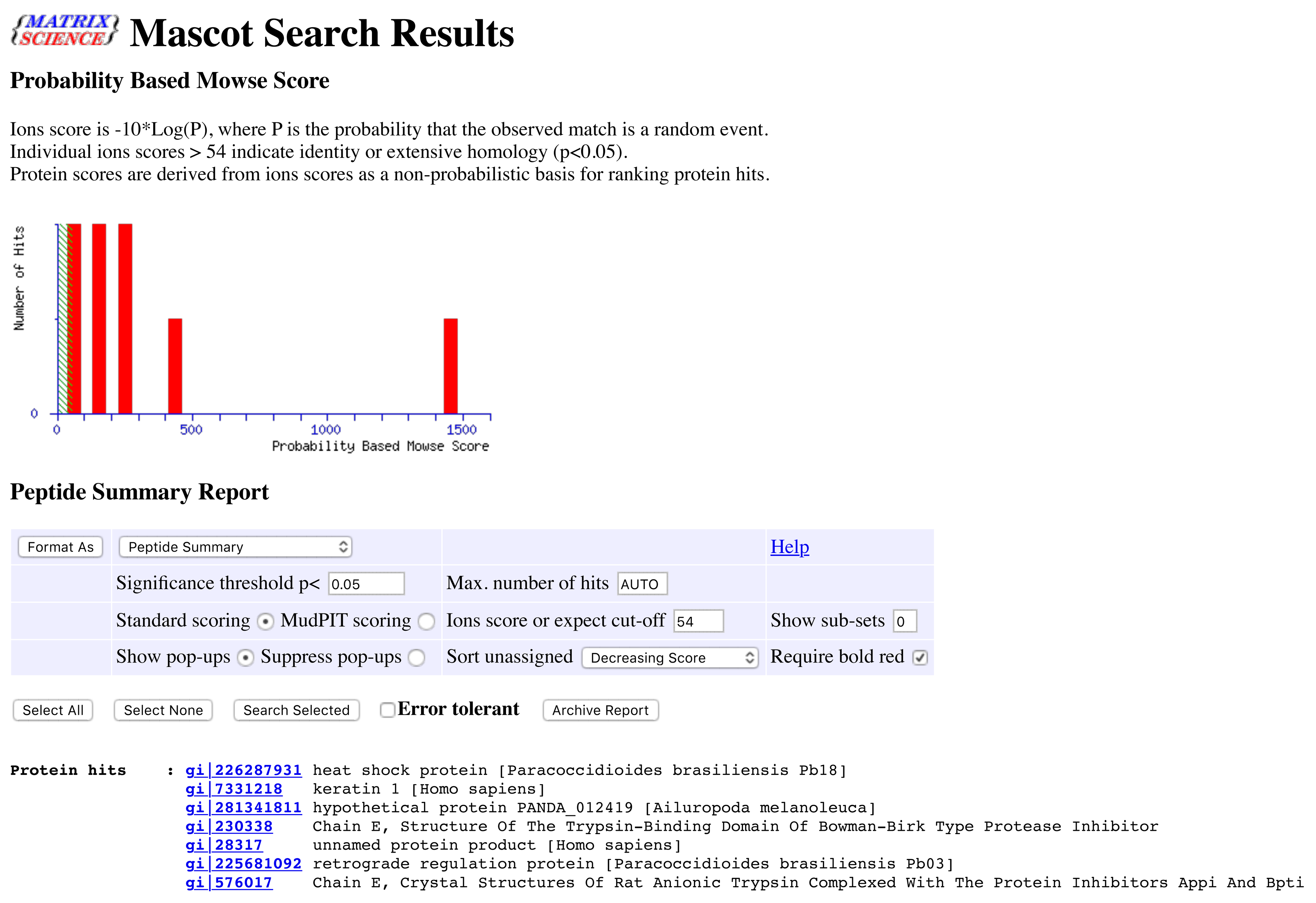

Supplement: S1 Fig — The 60-kDa band from P. brasiliensis was excised from the electrophoresis gel and digested in situ with trypsin. Peptides were extracted from gel and dried, resuspended in 50 μl 1% formic acid, centrifuged and transferred to HPLC vial. Spectrometry analyses were performed on an Agilent 6520 Q-TOF mass spectrometer equipped with an Agilent 1200 series liquid chromatograph and an Agilent Chip Cube LC-MS interface (1D nLC-MS-MS). Mascot (version 2.3; Matrix, United Kingdom) analysis was performed to identify peptides and to search for proteins in the NCBI nonredundant (nr) database. (TIF) [file pone.0162486.s001.tif]
