# Supplementary material for: Detrimental Effect of Fungal 60-kDa Heat Shock Protein on Experimental Paracoccidioides brasiliensis Infection
Source: PLoS One. 2016 Sep 6;11(9):e0162486. doi: 10.1371/journal.pone.0162486 (PMC5012565; doi:10.1371/journal.pone.0162486)
Supplement: S1 Table — (DOCX) [file pone.0162486.s002.docx]

**S1 Table. List of all proteins matched with at least one peptide with a significant score.**

| Accession | Description | Mass (Da) | Score | Query match | Coverage (%) |
| --- | --- | --- | --- | --- | --- |
| XP_010763632.1 | heat shock protein [Paracoccidioides brasiliensis Pb18] | 62,522 | 1457 | 141 | 43 |
| AAF60327.1 | keratin 1 [Homo sapiens] | 66,149 | 409 | 10 | 13 |
| EFB17395.1 | hypothetical protein PANDA_012419 [Ailuropoda melanoleuca] | 39,324 | 254 | 5 | 16 |
| 1TAB_E | Chain E, Structure Of The Trypsin-Binding Domain Of Bowman-Birk Type protease Inhibitor | 23,975 | 207 | 13 | 23 |
| CAA32649.1 | unnamed protein product [Homo sapiens] | 59,720 | 136 | 2 | 4 |
| EEH19376.2 | retrograde regulation protein [Paracoccidioides brasiliensis Pb03] | 62,712 | 129 | 3 | 4 |
| 1BRB_E | Chain E, Crystal Structures Of Rat Anionic Trypsin Complexed With The Protein Inhibitors Appi And Bpti | 24,467 | 63 | 1 | 5 |
